# Advantages of diffuse light for horticultural production and perspectives for further research

**DOI:** 10.3389/fpls.2015.00704

**Published:** 2015-09-04

**Authors:** Tao Li, Qichang Yang

**Affiliations:** ^1^Institute of Environment and Sustainable Development in Agriculture, Chinese Academy of Agriculture SciencesBeijing, China; ^2^Key Laboratory of Energy Conservation and Waste Management of Agricultural Structures, Ministry of AgricultureBeijing, China

**Keywords:** diffuse light, light distribution, photosynthesis, plant processes, horticultural production

## Abstract

Plants use diffuse light more efficiently than direct light, which is well established due to diffuse light penetrates deeper into the canopy and photosynthetic rate of a single leaf shows a non-linear response to the light flux density. Diffuse light also results in a more even horizontal and temporal light distribution in the canopy, which plays substantial role for crop photosynthesis enhancement as well as production improvement. Here we show some of the recent findings about the effect of diffuse light on light distribution over the canopy and its direct and indirect effects on crop photosynthesis and plant growth, and suggest some perspectives for further research which could strengthen the scientific understanding of diffuse light modulate plant processes and its application in horticultural production.

## Introduction

Solar light is composed of a diffuse and a direct component. Diffuse light arises from the scattering of light by molecules or larger particles in the atmosphere and comes from many directions simultaneously; direct light arrives in a straight line from the sun without being scattered (Iqbal, 1983). Plants use diffuse light more efficiently than direct light (Gu et al., 2002; Farquhar and Roderick, 2003; Gu et al., 2003; Alton et al., 2007; Mercado et al., 2009; Li et al., 2014a), it arises due to diffuse light creates a more homogeneous light profile in the canopy than direct light. Photosynthetic rate of a single leaf shows a nonlinear response to the light flux density (Marshall and Biscoe, 1980). High light level usually leads to photosynthetic saturation and decrease in light use efficiency (LUE), which often occur under direct light condition. Therefore, the direct light usually wastes photons by concentrating the light resource to only a fraction of all leaves, leading to a less efficient photosynthetic use of light by plant canopies (Gu et al., 2002). Diffuse light, however, effectively avoids the light saturation constraint by more evenly distributing light among all leaves in plant canopies, and leads to a more efficient use of light (Gu et al., 2002).

To investigate the effect of diffuse light on plant processes, many studies have been carried out by comparing plant responses on cloudy and clear days (Zhang et al., 2011; Urban et al., 2012); or by comparing the aftermath of volcanic and anthropogenic emissions (Gu et al., 2003; Mercado et al., 2009). Such type research implies not only a difference in the fraction of diffuse light, but also large differences in light intensity, and the subsequent changes in microclimatic parameters such as air and soil temperature, and vapour pressure deficit (VPD). These changes directly or indirectly influence plant processes. Recently diffuse glass has become available that increases the diffuseness of light without affecting light transmission in the greenhouse (Hemming et al., 2007, 2008, 2014). Studies have reported that such cover materials have a remarkable effect on plant growth and production (Hemming et al., 2007; Li et al., 2014a,b). Thus, the occurrence of diffuse glass not only provide a promising measure for improving horticultural production, but also offers an opportunity to explicitly explore the pure effects of diffuse light on light distribution over the canopy and its direct and indirect effects on crop photosynthesis and plant growth. In this review, we will discuss the effect of diffuse light on plant processes and its application in horticultural production, and subsequently point out the perspectives for further research.

## Diffusing the Incident Light Improves Spatial Light Distribution

**FIGURE 1:**
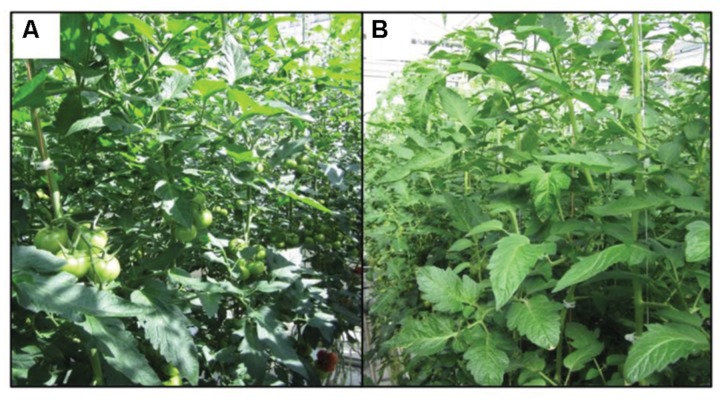
**Light distribution in tomato canopy in the conventional clear glasshouse (direct light, **A**) and diffuse glasshouse (diffuse light, B) on a clear day.** Light is more homogeneously distributed under diffuse light **(B)** compared with direct light **(A)** where many sunflecks in the middle and lower of the canopy. The photo was taken in Wageningen UR Greenhouse Horticulture, Bleiswijk.

Crop photosynthesis to a large extent correlates with the light profile within the canopy (González-Real et al., 2007; Niinemets, 2007; Sarlikioti et al., 2011a). In the vertical profile of the canopy, light intensity decreases exponentially from top to the bottom of the canopy, as described by the Beer-Lambert–Bouguer law (Chandrasekhar, 1950; Monsi and Saeki, 2005) of which light extinction coefficient can be used to quantify the vertical light distribution in the canopy. Diffuse light exhibits a lower extinction coefficient than direct light (Urban et al., 2012; Li et al., 2014a) although the effect depends on solar position (Morris, 1989). This indicates diffuse light penetrates deeper into the crop canopy. Such phenomenon occurred due to the properties of diffuse light that scatters in many directions and thus causes less shadow, while direct light either concentrates in a beam or casts a shadow in the canopy, which results in the upper leaves being brightly illuminated and lower leaves in deep shade, or strong sunflecks at a given canopy depth. In the horizontal profile of the canopy, diffuse light also results in a more homogeneous light distribution due to less sunflecks occur (Acock et al., 1970; Li et al., 2014a), which plays the most important role for crop photosynthesis enhancement under diffuse light (Li et al., 2014a). A general impression of light distribution in the canopy under direct as well as diffuse light condition has been given in **Figure 1**. Apart from light distribution, diffuse light also resulted in a lower leaf temperature and less photoinhibition of top leaves (Urban et al., 2012; Li et al., 2014a), which are correlated with the lower light absorption of the top leaves as well as fewer local peaks in light intensity occur under diffuse light, these are also benefit for crop photosynthesis.

Physiological and morphological properties of plant organs can be affected by their prevailing growth microclimate (Sultan, 2000; Niinemets, 2007). A homogeneous light distribution within the crop canopy under diffuse light gives rise to the question whether plant physiological and morphological acclimation occurs. Diffuse light penetrates deeper into the canopy; thus, the lower positioned leaves receive on average a higher light intensity which leads to a higher total nitrogen and chlorophyll content in the canopy, and consequently results in a higher leaf photosynthetic capacity in the lower of the canopy (Li et al., 2014a). Acclimation to diffuse light also includes acclimation of leaf morphology, which affects light interception and, consequently, photosynthesis (Pearcy et al., 2005). Li et al. (2014a) reported that tomato plants grown under diffuse light showed a lower specific leaf area (SLA) which indicates a thicker leaves, as well as a higher leaf area index (LAI) which mainly caused by a greater leaf width. A higher LAI is highly relevant for crop photosynthesis, as long as the fraction of light interception is also increased. For the mature crop under greenhouse condition, which often has a closed canopy, thus, the increased LAI under diffuse light has limited effect on canopy light interception and photosynthesis for mature crop (Li et al., 2014a).

## Diffusing the Incident Light Lessens the Variation of Temporal Light Distribution in the Canopy

In nature, temporal light distribution in the canopy is characterized by alternating periods of relatively high light followed by periods of background low light at a given point (sunflecks). Under these circumstances, a large fraction of CO_2_ assimilation may occur under transient light conditions. Stomata regulate carbon uptake of a leaf. In response to fluctuating light, stomata exhibit a dynamic response that is slower than the response of photosynthesis and fluctuating light itself, which may limit the CO_2_ assimilation under fluctuating light conditions (Pearcy et al., 2004; Lawson and Blatt, 2014). In greenhouses, the shadow and sunflecks generated by overstory leaves, leaf movement, greenhouse construction parts as well as equipment may exacerbate the variation of temporal light distribution. This may substantially limit crop photosynthesis compared to constant light intensities (Pearcy, 1990; Way and Pearcy, 2012). This variation in light intensity can be minimized when the incident light is made diffuse, which would consequently lead to less limitation on leaf photosynthesis, thus improving the canopy LUE (Li et al., 2014b).

Stomatal responses to dynamic light vary dramatically among species, from virtually no response to rapid stomatal responses, thereby resulting in different consequences for instantaneous leaf photosynthesis (Knapp and Smith, 1990; Vico et al., 2011), which may subsequently modulate the effect of diffuse light on canopy LUE. Li (2015) have tested the responses of two anthurium cultivars which have distinct stomatal properties to diffuse light. In cultivars where stomata respond strongly to fluctuations of photosynthetic photon flux density (PPFD), transient rates of photosynthesis and subsequently LUE increased under diffuse light in which stomatal conductance becomes relatively constant and less limiting for photosynthesis. For cultivars with relatively insensitive stomata to the fluctuations of PPFD, the effect of the homogeneous temporal distribution of PPFD on LUE was non-existing. In this context, additional to benefits of diffuse light associated with improved spatial light distribution, the stimulating effect of diffuse light on crop LUE can also depend on the dynamic response of stomatal conductance to incident PPFD at leaf level.

## Allowing More Light via Diffuse Cover Materials Stimulates Growth of Shade-Tolerant Pot Plants without Compromising Plant Quality

Even in northern countries, there are periods in summer with too high light levels for many shade-tolerant pot plants such as anthurium, bromeliads, and orchids. When excessive light energy is being absorbed by the light harvesting antennae at a rate which surpasses the capacity for photochemical and non-photochemical energy dissipation, this may lead to photoinhibition or photo-damage (Long and Humphries, 1994). In the long term, this could result in discoloring of leaves or even necrosis. Light damage occurs mostly as a result of prolonged exposure to excessive peaks in light intensity (Asada, 1999; Niyogi, 1999; Kasahara et al., 2002). Consequently, growers regularly apply shading in commercial production of many shade-tolerant pot plants in summer by closing a screen or having white wash on the greenhouse cover. However, shading often carries a penalty on potential crop growth as it is positively related to the amount of light that can be captured, which consequently reduces the LUE in the greenhouse production systems. When diffusing the incident light through cover materials, light in the greenhouse is more homogeneously distributed with less sunflecks, which decreases the extent of photoinhibition as well as local peaks in leaf temperature when global radiation is high (Li et al., 2014a). Therefore, the problem of discoloring of leaves or necrosis in shade-tolerant pot plants under relatively high light could be less when cultivated under diffuse light condition (Li et al., 2014b). Studies have suggested that increasing daily light integral under diffuse light not only accelerates plant growth but also improves plant ornamental quality with more compact plants (Li et al., 2014b; Marcelis et al., 2014). This may substantially contribute to the improvement of horticultural production.

## Perspectives for Further Research

Obviously, diffuse light has great advantageous for plant growth. However, detailed studies about the following aspects that closely related with diffuse light are lacking. Further exploring these aspects will strengthen the scientific understanding of diffuse light modulate plant processes as well as its application for crop production.

(a) The effects of diffuse light on crop photosynthesis could strongly differ between winter and summer light conditions. In winter, photosynthesis of the upper leaves is far from light saturation. With the same light intensity at leaf level, upper leaves have a higher rate of photosynthesis than lower leaves. Therefore, deeper penetration of light may have less effect on crop photosynthesis in winter (Sarlikioti et al., 2011b). Furthermore, light interception follows a seasonal pattern with on average, a lower fraction of light intercepted during summer than during winter because of changes in solar elevation (Sarlikioti et al., 2011a). The higher solar elevation in summer months results in an orientation of light rays more perpendicular to the plant canopy, resulting in a higher light penetration and lower interception. Therefore, seasonal variation of light intensity, directional light quality (diffuse or direct light) as well as solar position should be considered when exploring the effect of diffuse light on light distribution and crop photosynthesis.
(b) Measuring leaf photosynthesis is the basis for estimating canopy photosynthesis. Conventionally, only the adaxial side of the leaf is illuminated by the light source when measuring single leaf photosynthesis, this might result in minor error in estimating the canopy photosynthesis under diffuse light. This is because diffuseness of light may affect the fraction of light on the abaxial leaf surface, while the abaxial surface have a different photosynthesis light response curve than adaxial surface (Paradiso and Marcelis, 2012). Therefore, measurements of light absorption and photosynthesis light response curves on both the adaxial and abaxial side of leaves in the canopy in combination with functional–structural plant modeling might help to estimate these effects.
(c) Row crop systems are commonly used in horticultural and agronomic crops. This system facilitates crop management and allows higher light penetration inside the plant canopy. In this system, a fraction of light reaches the ground floor in the middle of the path (Stewart et al., 2003; Sarlikioti et al., 2011a), the reflection of light by the floor can be reused for photosynthesis. Furthermore, row orientation substantially affects canopy light interception (Borger et al., 2010; Sarlikioti et al., 2011a). These effects may differ between diffuse and direct light conditions.
(d) Light distribution and absorption is highly dependent on crop architecture (Falster and Westoby, 2003; Zheng et al., 2008; Sarlikioti et al., 2011b). Short and compact canopies may generate substantial leaf overlap and self-shading, therefore decreases the net amount of leaf area exposed to light, and consequently affect canopy light interception (Falster and Westoby, 2003). Plants also vary widely in leaf angle, leaf orientation, internode length, and leaf length to width ratio, these traits have a direct effect on light absorption and photosynthesis (Falster and Westoby, 2003; Sarlikioti et al., 2011b). However, detailed research about plant architecture modulates the effect of diffuse light on light distribution and canopy photosynthesis are lacking. Furthermore, LAI is a predominant factor for canopy light interception, at low LAI mutual shading of leaves within the canopy is small, thus light may readily penetrate deeper into the canopy, which probably decrease the potential effect of diffuse light.
(e) Fruit and vegetable quality is closely correlated with the pre-harvest growth condition. In open field and conventional clear greenhouses, fruit and vegetables often experience diurnal fluctuations or long-term exposure to direct sunlight, with associated high tissue temperatures. This may result in harvest disorders (i.e., sunburn), and heterogeneity of internal quality properties such as sugar content, tissue firmness, mineral content (Woolf and Ferguson, 2000). Fruit with different temperature histories will also respond differently to postharvest low temperatures (i.e., chilling injury) (Ferguson et al., 1999). The quality problems induced by sunlight exposure could be reduced if plants were grown under diffuse light where less fluctuations in temperature and light intensity occurs, detailed research in this aspect has not been reported so far.

## Conclusion

Diffuse light improves spatial light distribution in the crop canopy, thereby stimulating crop photosynthesis; the more uniform horizontal light distribution within the canopy plays the most important role for this effect. Diffuse light also lessens the variation of the temporal light distribution at any specific point in the canopy. However, its effect on plant growth depends on the dynamic responses of stomatal conductance to the incident light. Apart from the homogeneous light distribution, diffusing the incident light makes it possible to allow more light in the greenhouse which strongly stimulates crop growth of shade-tolerant pot plants without compromising plant quality. Although the available knowledge have clearly stated the advantageous of diffuse light for crop production, incorporating the seasonal light condition and solar position, plant architecture, crop management practices as well as the post-harvest product quality for further research will strengthen our understanding of the effect of diffuse light on plant processes.

## Conflict of Interest Statement

The authors declare that the research was conducted in the absence of any commercial or financial relationships that could be construed as a potential conflict of interest.
